# The role of the symbiotic microecosystem in cancer: gut microbiota, metabolome, and host immunome

**DOI:** 10.3389/fimmu.2023.1235827

**Published:** 2023-08-24

**Authors:** Xiaoyu Xue, Rui Li, Zhenni Chen, Guiyu Li, Bisheng Liu, Shanshan Guo, Qianhua Yue, Siye Yang, Linlin Xie, Yiguan Zhang, Junning Zhao, Ruirong Tan

**Affiliations:** ^1^ School of Pharmacy, Southwest Medical University, Luzhou, China; ^2^ Sichuan Institute for Translational Chinese Medicine, Sichuan Academy of Chinese Medical Sciences, State Key Laboratory of Quality Evaluation of Traditional Chinese Medicine, Sichuan Engineering Technology Research Center of Genuine Regional Drug, Sichuan Provincial Engineering Research Center of Formation Principle and Quality Evaluation of Genuine Medicinal Materials, Translational Chinese Medicine Key Laboratory of Sichuan Province, Chengdu, China; ^3^ Department of Radiation Oncology, Radiation Oncology Key Laboratory of Sichuan Province, Sichuan Clinical Research Center for Cancer, Sichuan Cancer Hospital and Institute, Sichuan Cancer Center, Affiliated Cancer Hospital of University of Electronic Science and Technology of China, Chengdu, China; ^4^ College of Food and Biological Engineering, Chengdu University, Chengdu, China; ^5^ Traditional Chinese Medicine Hospital Affiliated to Southwest Medical University, Classical Chinese Medicine Diagnosis and Treatment Center, Luzhou, China

**Keywords:** gut microbiome, cancer immunology, immunotherapy, tumor microenvironment, immunomodulation

## Abstract

The gut microbiota is not just a simple nutritional symbiosis that parasitizes the host; it is a complex and dynamic ecosystem that coevolves actively with the host and is involved in a variety of biological activities such as circadian rhythm regulation, energy metabolism, and immune response. The development of the immune system and immunological functions are significantly influenced by the interaction between the host and the microbiota. The interactions between gut microbiota and cancer are of a complex nature. The critical role that the gut microbiota plays in tumor occurrence, progression, and treatment is not clear despite the already done research. The development of precision medicine and cancer immunotherapy further emphasizes the importance and significance of the question of how the microbiota takes part in cancer development, progression, and treatment. This review summarizes recent literature on the relationship between the gut microbiome and cancer immunology. The findings suggest the existence of a “symbiotic microecosystem” formed by gut microbiota, metabolome, and host immunome that is fundamental for the pathogenesis analysis and the development of therapeutic strategies for cancer.

## 1 Introduction

Life, in the form of prokaryotes, first appeared on Earth approximately 3.8 billion years ago, while the earliest eukaryotic single-celled organisms emerged approximately 1.8 billion years ago. Evidence suggests that eukaryotes originated from the fusion and aggregation of prokaryotes into multicellular complexes, initially utilizing the genetic information of prokaryotes. This process led to the differentiation into animals and plants, with microorganisms playing a crucial role throughout the entire process. Hundreds of different types of microorganisms colonize the vertebrate intestine in a rather mutually beneficial interaction with the host (1–3). Among the gut microbiota, the members that play a dominant role are Firmicutes (Gram-positive bacteria without a true outer membrane) and Bacteroidetes (Gram-negative bacteria with an outer membrane) phyla (4–7). In healthy individuals, the gut microbiota maintains a dynamic balance between beneficial and opportunistic pathogenic bacteria (8). Moreover, these microorganisms actively contribute to the production of neurotransmitters, enzymes, and vitamins. For example, vitamins B and K produced by bacteria are involved in immune and metabolic functions (9–11). A dynamic equilibrium of the gut microbiota with beneficial bacteria predominating is the optimal condition for the gut microbiota. Disruptions to the dynamic equilibrium of the gut microbiota can lead to changes in the composition, amount, and activity of the microbial community (12, 13). These disruptions may occur due to factors such as age, dietary preferences, and illness. Consequently, the mucosal barrier may become impaired, leading to changes in cytokines and cell signaling, suppression of commensal bacteria and probiotic colonization, and increased proliferation of intestinal pathogens. These alterations can compromise both local and systemic immune responses (14). Damage to the mucosal barrier can result in the transfer of gut microbes to mesenteric lymph nodes (MLNs) and peripheral circulation, inducing Th17 and effector T-cell activation, promoting neutrophil infiltration, and activating local and systemic inflammatory responses (15).

**Figure 1:**
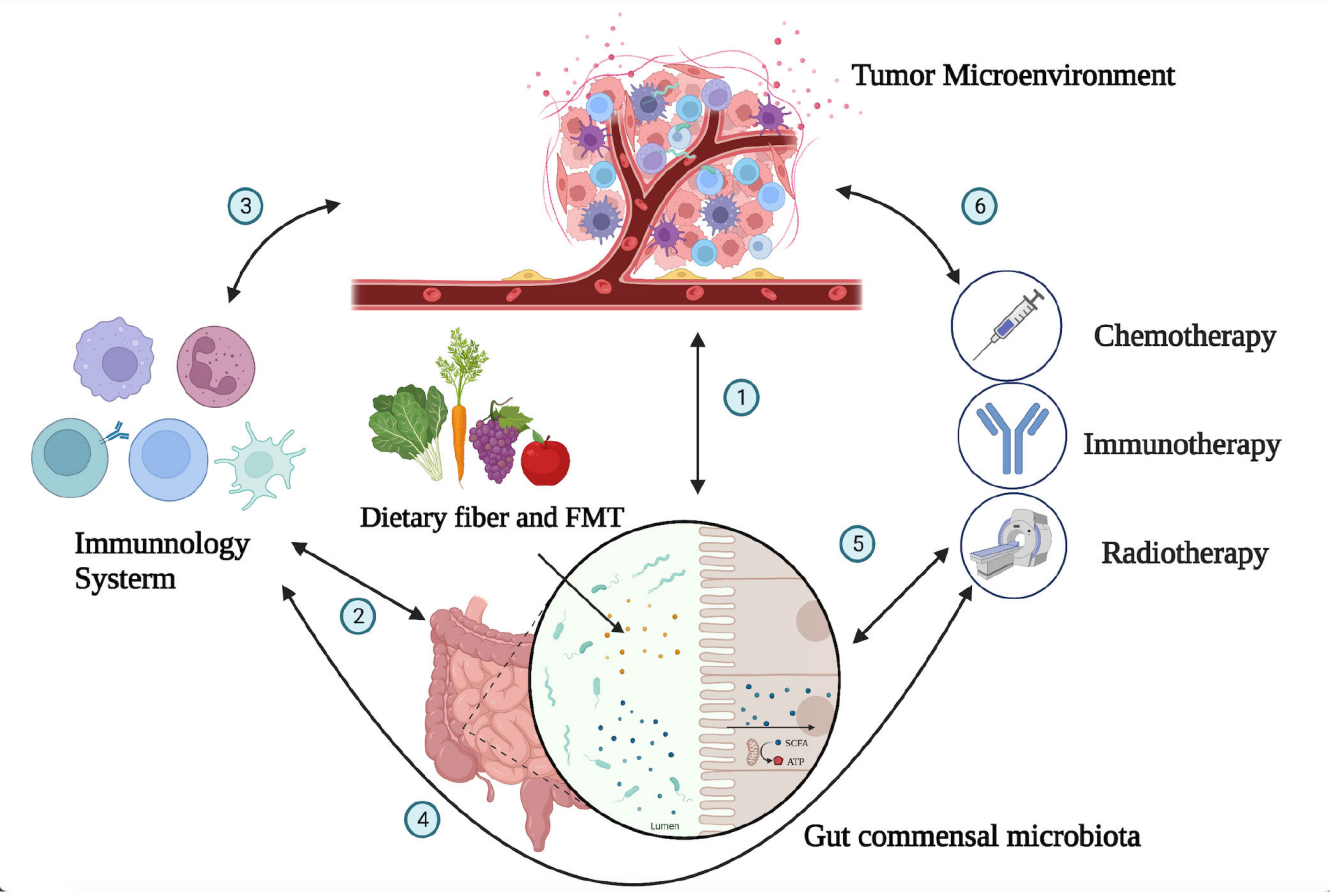
Interactions between the tumor microenvironment, gut microbiota, immune system, and immunotherapy. The gut microbiota can affect the occurrence and progression of tumors through a variety of mechanisms, including directly participating in the occurrence and progression of tumors; influencing the development and recognition of immune cells in the immune system, affecting the ability of immune cells to exert antitumor immunity; and collaborating with tumor treatment to improve treatment effectiveness. The numbers (1–6) in the figure demonstrate the dynamic and intricate connections among the tumor microenvironment, immune system, gut microbiota, and tumor treatment, with mutual influences on each other (1). The gut microbiota can influence tumor development through mechanisms such as modulating immune responses, affecting the growth and apoptosis of tumor cells, and regulating the expression of tumor-related genes. Conversely, changes in the tumor microenvironment can also impact the composition and function of the gut microbiota. The interplay between the tumor microenvironment and gut microbiota is a complex bidirectional relationship that holds significant implications for tumor development and treatment (2). The immune system can maintain immune balance in the gut by regulating the composition and function of the gut microbiota, and the gut microbiota can also influence the development and function of the immune system (5). The composition of the gut microbiota is related to individual responses to chemotherapy, radiation therapy, and immunotherapy. By modulating the composition and function of the gut microbiota, it may be possible to improve the effectiveness of tumor treatment and enhance patient survival rates and quality of life (3, 4, 6). The tumor microenvironment is closely related to the immune system, as cellular molecules and factors such as blood vessels within the tumor microenvironment can regulate immune system functions. Various treatment methods can impact the tumor microenvironment and immune system in different ways. Thus, there exists a complex interaction network among the immune system, tumor microenvironment, and tumor treatment.

The intestinal microbiota plays a complex and important role in the development and progression of tumors (16). The gut microbiota exhibits a bidirectional role in tumor development and progression. On one hand, certain bacteria can promote cancer by producing carcinogenic metabolites, inducing inflammation, and impairing immune responses. On the other hand, specific interventions such as probiotics and fecal microbiota transplantation (FMT) have demonstrated potential antitumor effects. Microbiota-driven carcinogenic mechanisms exhibit significant heterogeneity across different organs. For instance, specific bacteria have been implicated as pathogenic factors in gastric cancer, while changes in the intestinal microbiota and metabolites induced by dietary cholesterol drive NAFLD-HCC (non-alcoholic fatty liver disease-liver cancer) (17). However, the specific mechanisms involved in these processes remain largely unclear (18) (**Figure 1**).

## 2 The complicated bidirectional role of gut microbiome in cancer

### 2.1 Certain bacteria promote gastrointestinal cancer by inducing local inflammation and impairing the host immune response

**Figure 2:**
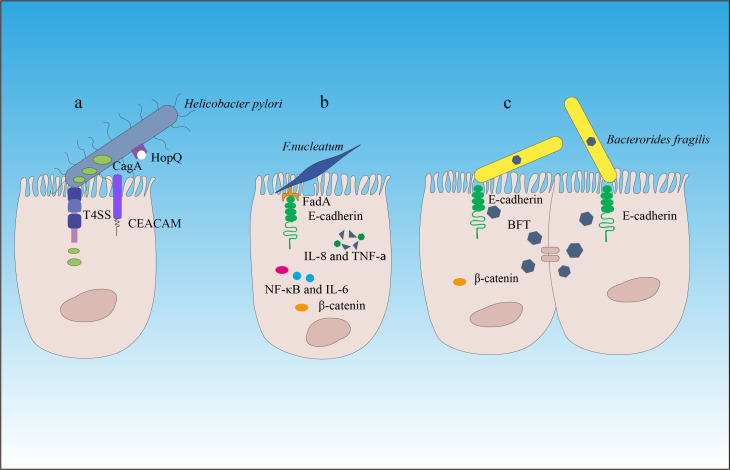
The carcinogenic mechanisms of *Helicobacter pylori*, *Fusobacterium nucleatum*, and *Bacteroides fragilis*. **(A)** *Helicobacter pylori* attaches to the gastric epithelial cells by binding to the carcinoembryonic antigen-related cell adhesion molecule (CEACAM) through the outer membrane adhesin HopQ (22). Then, through the type IV secretion system of CagA, peptidoglycan metabolites, and DNA, the effector protein CagA is directly delivered into the epithelial cells to exert carcinogenic effects. **(B)** Through its mechanism of action, *Fusobacterium nucleatum* contributes to the development and progression of colorectal cancer. **(C)** *Enterotoxigenic Bacteroides fragilis* (ETBF) produces toxins that target the tight junctions of intestinal epithelial cells, cleaving E-cadherin and promoting inflammation and destruction of the intestinal mucosal barrier. This induces chronic intestinal inflammation and tissue damage in colorectal cancer. β-catenin signaling alterations are a frequent target of cancer-associated microbes. Some microbes bind E-cadherin on colonic epithelial cells, with altered polarity or within a disrupted barrier, and trigger β-catenin activation. Other microbes inject effectors (e.g., CagA) that activate β-catenin signaling, resulting in dysregulated cell growth, acquisition of stem cell–like qualities, and loss of cell polarity.

If we consider the gut as the outer surface of the body (similar to the intestinal and skin surfaces), the gut microbiota belongs to the external symbiotic community. Clearly, it is not desirable to have such a situation inside cells or in peripheral blood and tissues (except in the case of individuals who develop symptoms after viral infections). In other words, the tolerance of the host immune system toward gut microbiota is mostly limited to the outer surface of tissues. To combat microbes that invade the internal tissues, the immune system needs to be activated or in a state of readiness (such as immune cells that are exposed to a large number of microbial antigens or immune receptor cells with diversity that can respond to microbial and mutated cell stimuli). This phenomenon is an important component of immune function because microbes from the outer surface of tissues can enter through various pathways. Without immune receptor cells in a state of readiness, we would struggle to defend against different types of microbial invasions. This also suggests that infants should be exposed to as many environmental microbes as possible soon after birth to stimulate the immune system and generate greater diversity, thereby maintaining a state of readiness. The presence of gut microbiota is crucial for maintaining the immune system’s readiness, and under normal circumstances, the two exist in a dynamic balance. However, if the quantity of a particular microbe or several microbes suddenly increases or remains at a consistently high level for a prolonged period, it can significantly impact the immune system. Apart from its pathogenicity (although current gut symbiotic microorganisms have evolved to have low virulence, a sharp increase in pathogenicity can occur when a large amount of the same strain becomes more virulent), it can greatly affect immune receptor diversity and immune spatial layout and directly contribute to the development of diseases. A classic example of a bacterial species that causes gastric cancer is *Helicobacter pylori* (19, 20). *H. pylori* overcomes the natural defense of the stomach by producing urease to neutralize the local acidic environment and using flagella, which have the ability to penetrate the mucous layer and interact with epithelial cells. Other characteristics contributing to the survival of *H. pylori* include its ability to adhere to the epithelium, produce catalase to neutralize hydrogen peroxide, and obtain nutrients (21). *H. pylori* is a cell toxin-associated antigen A (CagA) gene-positive bacterium that adheres to gastric epithelial cells by binding to the carcinoembryonic antigen-related cell adhesion molecule (CEACAM) through the outer membrane adhesin HopQ (22) and then delivers the effector protein CagA, peptidoglycan metabolites, and DNA directly to epithelial cells through a type IV secretion system (23). Translocated CagA protein is localized to the cell membrane and then undergoes tyrosine phosphorylation at the Epiya site, mediated by the SRC family tyrosine kinase. Phosphorylated CagA interacts with intracellular signaling molecules through the Src Homology 2 (SH2) domain, releases its activity regulation, and triggers pathological effects on gastric cancer (24–26) (**Figure 2**, **Table 1**).

**Table 1:** The role of commensal microbes in tumorigenesis and progression.

Cancer type	Microbiome	Proposed mechanism
Gastric cancer	*Helicobacter pylori*	*H. pylori* is a cell toxin-associated antigen A (CagA) gene-positive bacterium that adheres to gastric epithelial cells by binding to the carcinoembryonic antigen-related cell adhesion molecule (CEACAM) through outer membrane adhesin HopQ (22) and then delivers the effector protein CagA, peptidoglycan metabolites, and DNA directly to epithelial cells through a type IV secretion system (23).
Colorectal cancer	*Bacteroides fragilis*	*Enterotoxigenic Bacteroides fragilis* (ETBF) that produces the metalloprotease *Bacteroides fragilis* toxin (BFT) promotes inflammation and disrupts the intestinal barrier function by targeting the tight junctions of intestinal epithelial cells, which is associated with acute diarrhea and inflammatory bowel disease (27–29).
	*Escherichia coli*	*pks+ E. coli*-derived colicin causes DNA damage in colonic epithelial cells (30, 31).
	*Fusobacterium nucleatum*	*F. nucleatum* has been shown to bind to host epithelial and endothelial cells through FadA adhesin and induce a series of inflammatory reactions mediated by NF-κB and IL-6 (32–34).
Breast cancer	*Clostridiaceae*, *Faecalibacterium*; *Ruminococcaceae*, *Dorea* Lachnospiraceae	The gut microbiota may affect breast cancer risk and may do so through estrogen-independent pathways (35–37).
	*Fusobacterium nucleatum*	Gal-GalNAc levels increase as human breast cancer progresses, and that occurrence of *F. nucleatum* gDNA in breast cancer samples correlates with high Gal-GalNAc levels (38).
Lung cancer	*Veillonella* and *Megasphaera* (39), Gram-negative bacilli *Escherichia coli*, *Haemophilus influenzae*, *Staphylococcus* spp., *Candida albicans* (40), *Streptococcus* (41)	Local microbiota provoke inflammation associated with lung adenocarcinoma by activating lung-resident γδ T cells (42).
Skin cancer	*S. aureus* (43)	In squamous cell carcinoma (SCC), *S. aureus* might promote tumor cell growth via modulation of hBD-2 expression (43).
	*Corynebacterium* (44, 45)	*Corynebacterium* species might affect the development of MM (malignant melanoma) through an IL-17-dependent pathway (44, 45).
Pancreatic cancer	*Fusobacterium nucleatum* (46, 47), *Granulicatella adiacens* (47)	The possible association of tumor *Fusobacterium* species status with epigenetic alterations, such as MLH1 methylation and CpG island methylator phenotype (CIMP) in pancreatic cancer.
	Proteobacteria (48, 49)	Proteobacteria lead to T-cell anergy in a Toll-like receptor-dependent manner, accelerating tumor progression (49).
	*Malassezia globose* (50, 51)	Contributes to tumorigenesis, tumor growth, and gemcitabine resistance via mannose-binding lectin-C3 axis (50, 52)
Cervical cancer	Proteobacteria*, Parabacteroides, Escherichia-Shigella, Roseburia* (53)	The role of *Prevotella* in altering host immunity by modulating immunologic pathways may also be linked to cervical cancer risk and treatment outcomes (54).
	*Prevotella, Porphyromonas, Dialister* (54)	
Esophageal cancer	*Fusobacterium nucleatum* (55)	Contributes to tumor infiltration of Treg lymphocytes in a chemokine (especially CCL20)-dependent fashion, promoting aggressive tumor behaviors (55).
	*Campylobacter* (56)	*Campylobacter* in esophageal adenocarcinoma progression might mimic that of *Helicobacter pylori* in gastric cancer (57, 58).
Hepatocellular carcinoma	*Clostridium* (59)	Inhibition of DCA production and modulation of gut microbiota are effective in preventing tumorigenesis in HCC; however, *Clostridium* metabolizes bile acid into DCA, thus increasing the serum level of DCA (deoxycholic acid) 0p’in HCC (59, 60).
	*Salmonella typhi*	*Salmonella typhi* strains that maintain chronic infections secrete AvrA, which can activate epithelial β-catenin signaling (61–63).
	*Bacteroides* and *Ruminococcaceae*	*Bacteroides* and *Ruminococcaceae*, can contribute to the development of hepatocellular carcinoma (HCC) by exacerbating hepatic inflammation, accumulating toxic compounds, and causing liver steatosis (64).

HCC, hepatocellular carcinoma; DCA, deoxycholic acid.

The dysbiosis of the gut microbiota is also closely related to the occurrence of colorectal cancer (CRC) (27, 65, 66). Previous research has revealed that the prevalence of several bacterial groups, such as *Bacteroides fragilis* and *Fusobacterium nucleatum*, in the fecal microbiota of CRC patients was higher than that of normal individuals (67–69). *F. nucleatum* has been shown to bind to host epithelial and endothelial cells through FadA adhesin and induce a series of inflammatory reactions mediated by Nuclear Factor-kappa B (NF-κB) and interleukin (IL)-6 (32–34). Hong et al. (70) have found that *F*. *nucleatum* abundance correlated with high glucose metabolism in patients with CRC. *F. nucleatum* induces a dramatic decline of m^6^A modifications in CRC cells and patient-derived xenograft (PDX) tissues by downregulating an m^6^A methyltransferase, METTL3, contributing to the induction of CRC aggressiveness (71). In addition, *F. nucleatum* can also inhibit the cytotoxic functions of tumor-infiltrating lymphocytes and natural killer (NK) cells by binding to the inhibitory immune receptor TIGIT through another adhesin, Fap2, thereby suppressing immune surveillance (33, 72). In addition, *F. nucleatum* may contribute to epithelial–mesenchymal transition (EMT), so it is tightly associated with cancer cell invasion, suppression of antitumor immune responses, stemness, and treatment resistance (73).

*Enterotoxigenic Bacteroides fragilis* (ETBF) that produces the metalloprotease *Bacteroides fragilis* toxin (BFT) promotes inflammation and disrupts the intestinal barrier function by targeting the tight junctions of intestinal epithelial cells (IECs), which is associated with acute diarrhea and inflammatory bowel disease (74), thereby inducing chronic inflammation and tissue damage in CRC (27, 28, 75–77). In addition, ETBF has been found to be enriched in the gut microbiota of CRC patients, and its enrichment is associated with poor prognosis of CRC (29, 78). ETBF, which is enriched in some human CRCs, can stimulate E-cadherin cleavage via BFT, leading to β-catenin activation (79). Studies have also found that ETBF plays an important role in promoting CRC through the Toll-like receptor 4 (TLR4)-Nuclear Factor of Activated T-cells 5 (NFAT5)-dependent upregulation of Jumonji domain-containing protein 2B (JMJD2B) levels in stem cell regulation (80, 81).

### 2.2 Gut microbiota also promotes extraintestinal cancers through bacterial translocation and production of bioactive molecules into circulation

Organs outside of the gastrointestinal tract are also remotely affected by the gut microbiota’s carcinogenic effects. A study found that certain gut microbiota, such as the *Bacteroides* and *Ruminococcaceae*, can contribute to the development of hepatocellular carcinoma (HCC) by exacerbating hepatic inflammation, accumulating toxic compounds, and causing liver steatosis (64). Obese and lean people have substantially distinct gut microbiota compositions, especially in terms of the proportion of bacteria that produce pro-inflammatory lipopolysaccharides (LPSs). In accordance with thorough experimental investigations, transplanting the microbiota of healthy individuals into obese mice can reduce steatosis while causing hepatic steatosis in mice that are fed normally (82).

Since symbiotic microbiota are frequently stable in the gastrointestinal system, researchers posed an essential question: where do tumor-related bacteria in remote organs come from? Geller et al. proposed that pancreatic ductal adenocarcinoma (PDAC)-related bacteria can retrogradely originate from the gastrointestinal tract (48, 83). Pushalkar et al. (49) provided evidence of bacterial migration from the gut to the pancreas, as well as a time-dependent association between gut dysbiosis and Kras activation in PDAC. Vitiello et al. (83) also found that gut dysbiosis can directly promote oncogenic signaling in the pancreas. Accordingly, dysbiosis and mislocalization of the gut microbiota have been associated with the onset of pancreatic and liver cancers, which is in line with the theory that the intestine, liver, and pancreas maintain continuous interactions. Microbiota can also indirectly affect tumor progression through the production and metabolism of bioactive molecules, which may reach tumors and metastatic sites via systemic circulation, such as bacterial LPS, which can enter the bloodstream and affect tumor formation in tissues far from the gastrointestinal tract (84). Deoxycholic acid (DCA) and lithocholic acid (LCA) can cause DNA damage by increasing the production of reactive oxygen species (ROS), leading to cell senescence, chronic inflammation, and tumorigenesis (85–87).

### 2.3 Colonization of certain strains of probiotics in gut may have an antitumor effect through modulating the immune system and reducing inflammation

Maintaining a healthy and balanced gut microbiota can help suppress tumor development. The symbiotic microbiota benefits from the nutrient-rich environment in the gut, where the microbiota will produce hundreds of proteins and metabolites that regulate key host functions, including nutrient processing, energy balance maintenance, and immune system development. The gut microbiota has complex impacts on the growth of tumors, and probiotics that colonize the gut can influence these processes by enhancing immune responses to antigens and antibodies, inhibiting monocyte proliferation, and upregulating anti-inflammatory cytokines such as IL-10 and IL-12. The gut microbiota contributes to reducing pro-inflammatory cytokines such as IL-1β and IL-6, exhibiting effective anti-inflammatory activity. Goldin et al. have found that probiotics play an important role in preventing CRC (88, 89).

Lactic acid bacteria and *Bifidobacterium* are involved in regulating pH and bile acid processes (90). Inhibiting the activity of glucoside and nitrite reductase and decreasing the synthesis of carcinogenic chemicals are two effects that *Lactobacillus acidophilus* can have on the gut’s putrefactive bacteria. Lactic acid bacteria and *Bifidobacterium* can physically degrade potential carcinogens and their metabolites, such as heterocyclic amines (91–93), nitrosamines, and aflatoxins, thereby inhibiting the development of various cancers such as gastric, esophageal, liver, colon, and bladder cancers (94). In female mice receiving subcutaneous injection of breast cancer cells (4T1), giving them plant-derived *Lactobacillus* rich in selenium nanoparticles (SeNPs) was shown to induce effective immune responses by increasing levels of pro-inflammatory cytokines interferon gamma (IFN-γ), tumor necrosis factor alpha (TNF-α), and IL-2 and increasing NK cell activity, significantly inhibiting tumor development and increasing survival rates compared to mice receiving only plant-derived *Lactobacillus* or control model mice (95). Interestingly, whether through preventative use of milk fermented with the *Lactobacillus* CRL431 strain of probiotics or starting use of milk fermented with the CRL431 strain of probiotics after injection of breast cancer cells (4T1), giving probiotics delayed or prevented tumor development compared to mice injected with tumor cells (96).

The most common treatment for non-muscle-invasive bladder cancer is transurethral resection of bladder tumor (TURBT) (97), followed by single-dose intravesical immunotherapy with Bacillus Calmette-Guérin (BCG), which is an effective method for preventing bladder cancer recurrence and progression after bladder surgery (98–100) (**Table 2**). BCG works by inducing nonspecific immune reactions and mediating antitumor effects through the activation of inflammatory responses. CD4^+^ and CD8^+^ lymphocytes, NK cells, granulosa cells, giant cells, and dendritic cells (DCs) may be involved (118–121). In a mouse model of subcutaneously implanted CT26 colon cancer cells, it was found that pretreatment with *Lactobacillus plantarum* (KC836552.1) significantly reduced tumor growth, prolonged survival time, activated innate immunity, and increased the intratumor levels of CD8^+^ T and NK cells (122). Although the antitumor effect of *Lactobacillus rhamnosus* (JF414108.1) on colon cancer is not clear, it has been shown to be more effective than BCG in reducing bladder cancer recurrence rates. *L. rhamnosus GG* (LGG) can recruit large numbers of neutrophils and macrophages to the tumor site, thereby promoting tumor regression (104).

**Table 2:** Microbes inhibit tumor development.

Microbiome	Inhibition of cancer
*Cutibacterium acnes*	After intratumoral injection with *C. acnes*, the growth of melanoma cells was inhibited through the induction of Th1 type cytokines such as IL-12, tumor necrosis factor alpha (TNF-α), and interferon gamma (IFN-γ) (101).
*Staphylococcus epidermidis*	6-N-hydroxyaminopurine (6-HAP) produced by *Staphylococcus epidermidis* selectively inhibits the proliferation of tumor cell lines, and intravenous injection of 6-HAP inhibits the growth of B16F10 melanoma in mice (102).
Lactobacillus rhamnosus GG	Oral intake of lipoteichoic acid from *Lactobacillus* reduces skin cancer risk (103).
	*L. rhamnosus GG* (LGG) can recruit large numbers of neutrophils and macrophages to the tumor site, thereby promoting tumor regression (104, 105).
Lactic acid bacteria	Regulating pH and bile acid processes (90)
	Lactic acid bacteria could bind heterocyclic amines and other food-derived mutagens, reducing their activity (106).
	Anticancer activity of lactic acid bacteria polysaccharides is associated with the stimulation of immune cells, primarily lymphocytes T and B, macrophages, and NK cells, releasing interleukins (107).
	Lactic acid bacteria degrade potential carcinogens and their metabolites, such as heterocyclic amines, nitrosamines, and aflatoxins, through physical binding, thereby inhibiting the development of various cancers such as gastric cancer, esophageal cancer, liver cancer, colon cancer, and bladder cancer (91–93, 95, 108–110).
	Lactic acid bacteria can significantly reduce proangiogenic genes, including the expression of the VEGF gene, and stimulate the expression of Anti-angiogenic genes (111).
	Anticancer activity of lactic acid bacteria exopolysaccharides is associated with their antiproliferative and proapoptotic properties (112).
Bacillus Calmette-Guérin	Intravenous immunotherapy with Bacillus Calmette-Guérin (BCG) after TURBT for non-muscle-invasive bladder cancer can effectively prevent bladder cancer recurrence and progression (98–100).
*Bifidobacterium*	Competition with putrefactive and pathogenic microbiota; improvement of the host’s immune response (113).
	In the C57BL/6-B16 model, *Bifidobacterium* administration *per se* has a beneficial effect on CT26 tumor inhibition (114).
	Oral administration of *Bifidobacterium* alone improved tumor control to the same degree as programmed cell death protein 1 ligand 1 (PD-L1)–specific antibody therapy (checkpoint blockade), and combination treatment nearly abolished tumor outgrowth (115, 116).
*Streptococcus thermophilus*	The tumor-suppressive effect of *S. thermophilus* is mediated at least by the secretion of β-galactosidase (117).

In addition, certain specific types of bacteria can alter immune responses by promoting the development of certain subtypes of lymphocytes. For example, segmented filamentous bacteria (SFB) can induce the production of IL-17 and IL-22, which is beneficial for the production of Th17 cells in mice (123–125). When using a recombinant germ-free (GF) mouse model with the gut microbiota of gas-producing Clostridia and fragile bacteria, the fragile bacteria promote the production of regulatory T cells (Tregs) and IL-10-secreting T cells by binding surface polysaccharide A (PSA) to TLRs on Treg surfaces (126, 127). Sivan et al. compared melanoma growth in mice harboring different commensal microbiota and observed differences in spontaneous antitumor immunity that were abolished after mouse cohousing or FMT. The 16SrRNA sequencing of mouse feces identified *Bifidobacterium* that may cause differences in tumor growth and antitumor immunity in mice; studies have also found that the *Bifidobacterium* can enhance DC function, leading to the initiation and clustering of CD8^+^ T cells in the tumor microenvironment (TME), thereby improving the antitumor immune function (115, 128, 129).

### 2.4 Metabolites produced by gut microbiota could influence the function of the intestinal epithelial barrier as well as the immune response

Intestinal symbiotic microbiota can also affect host immunity through metabolites. Many metabolites produced by microbes come from undigested or partially digested dietary fibers in the body. The metabolites that play a major role in gut and human health are short-chain fatty acids (SCFAs), which link host nutrition to gut homeostasis. Through a variety of mechanisms, SCFAs regulate IECs’ functions, including proliferation and differentiation, and the subpopulations of intestinal endocrine cells that affect gut motility, intestinal barrier performance, and host metabolism. Recent studies have demonstrated improved intestinal barrier and immunological regulatory activities of SCFAs, particularly butyrate (130–132). Butyrate not only promotes the production of iTregs as a Histone Deacetylase (HDAC) inhibitor, which is crucial for intestinal balance, but also promotes iTreg differentiation by enhancing fatty acid oxidation (FAO) (133, 134). In CRC patients, there is a significant reduction in butyrate-producing bacteria, and intestinal tumor cells treated with butyrate-producing Clostridia show reduced proliferation and increased apoptosis. Butyrate-producing bacteria can suppress the development of intestinal tumors by regulating Wnt signaling and gut microbiota, indicating potential therapeutic efficacy against CRC (135, 136). In addition to SCFAs, other metabolites produced by microbes, such as lipoteichoic acid (LTA) and secondary bile acids, have a dual effect on tumor development, while lysophosphatidic acid and secondary bile acids promote tumor development (137).

## 3 The impact of gut microbiota on tumor immunity

### 3.1 The influence of gut microbiota on immunity

**Figure 3:**
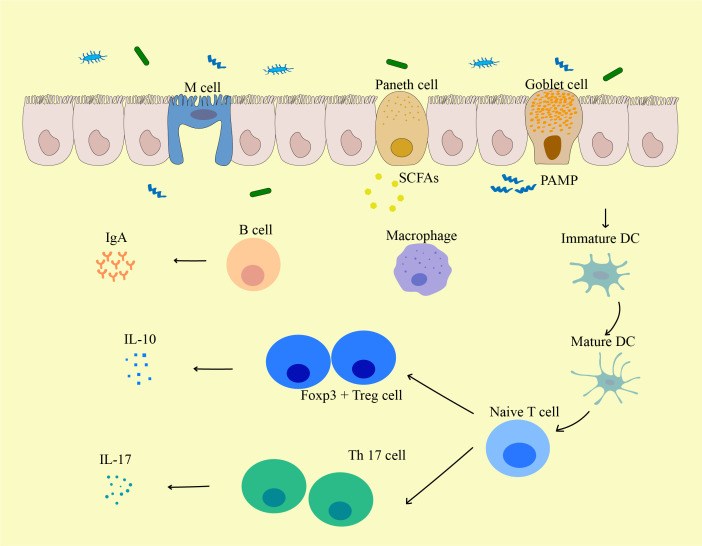
The influence of gut microbiota on the biological function of immune cells. Pathogen-Associated Molecular Pattern (PAMPs) from the gut induce the activation and maturation of antigen-presenting cells (APCs) including DCs. These APCs may then traffic to mesenteric lymph nodes to mediate the maturation of lymphocytes, and local DCs may be activated by bacterial metabolites (such as Short-chain fatty acids (SCFAs)) or bacteria themselves to migrate to mesenteric lymph nodes. Matured DCs further activate naive T cells to differentiate into effector T cells, Tregs, or Th17 cells, which can migrate back to the gut mucosa or systemic circulation. For local immune responses, Tregs secrete IL-10 and act on producing a local anti-inflammatory cytokine environment. Th17 cells secrete cytokines including IL-17 that induce IECs to form tight junctions and secrete antimicrobial proteins, and IL-17 can further lead to the release of other pro-inflammatory cytokines. PAMP, pathogen-associated molecular pattern; M cell, Microfold cell; SCFA, Short-chain fatty acids; DC, Dendritic cells.

Gut microbiota plays a crucial role in the development and maintenance of the host immune system (**Figure 3**) (138, 139), and the types and distribution of gut microbiota can directly and indirectly affect the immune system and are closely related to the occurrence of various diseases (140). By modifying the gut metabolome, the variety and location of gut microbiota might affect the repertory of gut or peripheral blood immune cells, impairing the immune system’s ability to recognize microorganisms or tumor cells. In the early stages of life, gut microbiota shapes the immune system, and changes in gut microbiota can affect the development and maturation of the immune system later in life (22). Therefore, the diversity of the gut microbiota is crucial for the establishment of the immune regulatory network.

The diversity of T-cell receptors (TCRs) in an immune repertoire with high levels of diversity is a key determinant of the host’s ability to resist various environmental pathogens (141). TCR diversity results from the random rearrangement of TCR gene segments and the fusion of TCRα and TCRβ chains during thymic T-cell maturation. As a response, the immunological repertoire can be created at different molecular levels from TCRs (142). The microbiota of a host is a complex community of microbial species that can form tissue-specific T-cell responses in mucosal tissues such as the respiratory tract, gastrointestinal tract, and urogenital tract and can induce CD4^+^ T cells to differentiate into various T helper cell subtypes, such as peripheral Foxp3^+^ Treg and Th17 cells in the gut (143). Early in life, the gut microbiota controls the location of innate lymphoid cells that express the transcription factor Promyelocytic Leukemia Zinc Finger (PLZF) in the thymus. The symbiotic microbiota’s extracellular signals that impact the gut immunoglobulin pool control the early B-cell lineage in the gut mucosa (144). The diversity of gut microbiota during early life colonization is critical for establishing an immune regulatory network that prevents the induction of mucosal immunoglobulin E (IgE), which is associated with allergy susceptibility (145). Though being limited to the neonatal stage in mice, TLR5-mediated negative selection of flagellated colonizing bacteria is an important process that determines the composition of the gut microbiota (146), and perturbations in this gut–thymus communication during early life can affect adult susceptibility to disease (136).

Microbes contribute to shaping the immune system (147), and GF mice, which lack a gut microbiota, are thought to have severe immune defects, including a lack of intestinal mucosal layer. Other defects include altered secretion of IgA and decreased size and function of Peyer’s patches and intestinal draining lymph nodes (mLNs) (148, 149). Intraepithelial lymphocytes (IELs) of type αβ and γδ in GF mice are substantially lower compared to typically colonized animals and can be significantly increased upon recolonization (150), and the development of thymic innate lymphoid cells in GF mice is impaired and lacks microbial ligands leading to defective TCR signaling (151). During colonization with the ubiquitous gut microbe *B. fragilis*, a bacterial polysaccharide (PSA) directs the cellular and physical maturation of the developing immune system. Compared with GF animals, PSA-mediated immune regulation during *B. fragilis* colonization includes the correction of systemic T-cell defects and Th1/Th2 imbalances and guidance of lymphoid organogenesis, and *B. fragilis* PSA mutants are unable to restore these immune functions. PSA expressed by intestinal DCs can activate CD4^+^ T cells and induce corresponding cytokine production (127). The thymic homeostasis of developing PLZF-expressing cells is likewise influenced by the gut microbiome. Specific developmental periods are crucial for the effect of gut–thymus communication on the thymus’s innate lymphoid cell development. Early-life antibiotic treatment can cause permanent damage to PLZF+ innate lymphoid cells in the thymus, while antibiotic treatment during adulthood does not result in damage (152). Colonization with a human commensal bacterium, segmented filamentous bacteria (SFB), but lacking PSA, can restore the thymic development of PLZF+ innate lymphoid cells in GF neonatal mice. Early in life, plasmacytoid DCs are influenced by the microbiota and migrate from the colon to the thymus to regulate the homeostasis of PLZF+ cells. More importantly, disturbance of thymic PLZF+ cells due to changes in the gut microbiota during early life affects susceptibility to diseases during adulthood, and this study identified a communication pathway between gut microbiota and thymic lymphoid cells during the neonatal period that regulates the host’s susceptibility to immunological diseases in later life (152). SFB is one of the few identified microbiota-specific TCRs (123). It induces polarization of Th17 cells, and these Th17 cells have a specific TCR for the SFB antigen (153, 154). Zegarra et al. found that early-life colonization of the gut commensal microbiota causes DCs in the intestine to transport microbial antigens to the thymus, followed by the induction of the expansion of microbiota-specific T cells. Once they enter the periphery, microbiota-specific T cells have the potential to become pathogenic or resist relevant pathogens. In this way, developing microbiota shapes and expands the thymic and peripheral T-cell repertoire, enhancing recognition of gut microbiota and pathogens (155). Stappenbeck et al. (156) examine the interactions between the gut microbiota, the small intestinal epithelium, and the villus’s mesenchymal microvascular network, and they show that the microbiota plays a key role in constructing this microvascular network, and that this regulation depends on a central component of the gut’s innate immune system: the Paneth cell. A conserved bacterial ligand produced by vitamin B synthesis activates the distinct innate T cells known as mucosal-associated invariant T (MAIT) cells, which link innate and adaptive immunity and are crucial in the body’s response to bacterial and viral infections. The development of thymic MAIT17 cells depends on 5-(2-oxopropylideneamino)-6-d-ribitylaminouracil (5-OP-RU) produced by commensal bacteria on the mucosal surface (157, 158).

The gut mucosa consists of IECs and IELs, including Paneth cells that secrete antimicrobial peptides and goblet cells that produce mucus. The gut-associated lymphoid tissue (GALT) is the most significant component of the human immune system (159). The submucosa of the mucosa contains Peyer’s patches and various immune cells, such as antigen-presenting cells, innate lymphoid cells, CD4^+^ T cells, CD8^+^ T cells, and B cells (160). Plasma cells in the lamina propria secrete IgA into the intestinal lumen, which binds to various components of microorganisms, dietary and luminal antigens, preventing harmful antigens from directly interacting with the host immune system (161). Fermentation products of commensal microbiota such as butyrate can induce differentiation of colonic Tregs in mice (134). Bacterial metabolites or the bacteria themselves can activate local DCs (162), which migrate to draining lymph nodes and activate naive T cells to become effector T cells. These T cells can subsequently return to the intestinal mucosa or enter the systemic circulation as Treg or Th17 cells (163). Specific metabolites or bacterial by-products can shape DCs to favor a Treg or Th17 phenotype. Some bacterial metabolites can directly enter the bloodstream and further modulate the systemic immune system (164). To prevent infections and preserve immunological homeostasis, the gut microbiota and the host immune system continually interact and impact each other. Commensal bacteria can signal to immune cells in GALTs and MLNs via pattern recognition receptors (PRRs), such as TLRs that recognize pathogen-associated molecular patterns (PAMPs), such as bacterial LPS and flagellin, to stimulate downstream immune responses (164–167). Sensing the commensal microbiota through the TLR-MyD88 signaling pathway triggers several responses that are critical for maintaining host microbial homeostasis. MyD88-dependent bacterial signaling is required for the induction of epithelial antimicrobial proteins such as RegIIIγ (168, 169). Subsequently, the microbiota induces the repair of damaged IECs through a MyD88-dependent process (170).

Allied with the microbiota, the gut microbiome can affect the immune system by releasing different metabolites into the bloodstream, including SCFAs (171). SCFAs are ligands for HDAC inhibitors and G protein-coupled receptors (GPCRs); SCFA-driven HDAC inhibition tends to express tolerant and anti-inflammatory cell phenotypes, playing an important role in maintaining immune balance (172). SCFAs have been shown to enhance epithelial barrier function and immunological tolerance, as well as promote gut homeostasis through particular mechanisms such as increased mucin secretion in intestinal villi, inhibiting NF-κB, activating inflammatory vesicles and producing IL-18, and increasing the secretion of IgA from B cells. SCFAs can also reduce antigen-presenting cells and directly or indirectly act on local or resident antigen-presenting cells in other organs, such as in the brain and lungs, thereby reducing neuroinflammation and inflammation associated with allergic airway diseases (173, 174). SCFAs also play an important role in the functional development of microglia; microglial dysfunction in GF mice can be rescued by SCFA treatment (175). GPR109A (encoded by Niacr1) is a butyrate receptor in the colon and a receptor for niacin, which is produced by the gut microbiota and inhibits gut inflammation. Studies have found that GPR109A signaling promotes the anti-inflammatory properties of colonic macrophages and DCs, enabling them to induce the differentiation of Tregs and IL-10-producing T cells (176). The most effective anti-inflammatory property of SCFAs is probably their ability to promote the activity of Tregs, which suppress the activity of effector T cells. Acetate and propionate can stimulate the expansion of colonic Tregs (cTregs) that already exist, while butyrate and propionate can promote the *de novo* differentiation of naive T cells into Tregs (82, 177). Numerous additional metabolites created by the gut microbiota from dietary components also play a significant role in immunity. For instance, the gut microbiota can use arginine, another amino acid, to generate metabolites that regulate the immune system. Polyamines, such as putrescine (a diamine, N2), spermidine (N3), and spermine (N4), which are derived from arginine and produced and secreted by gut bacteria, are present in every living cell and play important roles in gene expression and proliferation. Oral polyamine intake can enhance the development and maintenance of intestinal mucosa and resident immune cells (178, 179).

### 3.2 The impact of gut microbiota on the tumor microenvironment

**Figure 4:**
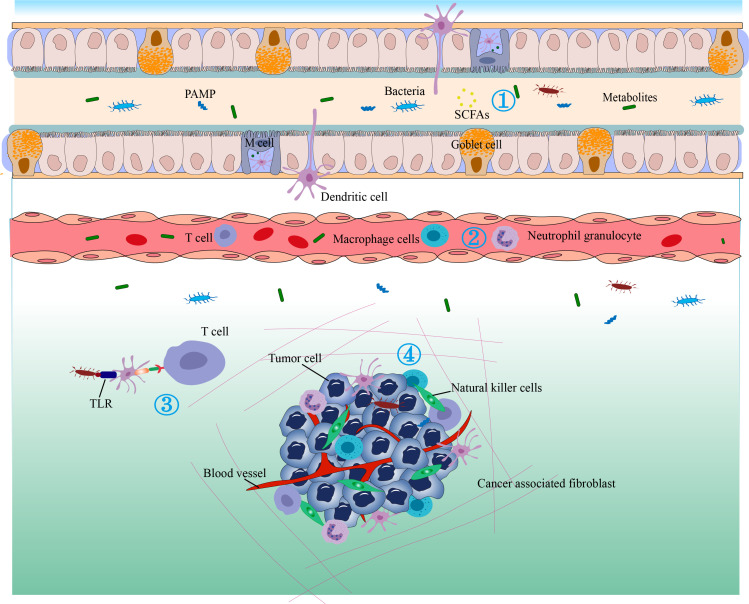
The antitumor immunity and immunotherapy effects of the gut microbiome. 1) Bacterial metabolites that enter the circulation can regulate gene expression in various cells. 2) Cytokines can be released in response to microbial stimulation in the GALT and may enter the circulation to regulate the immune function of downstream systems and stimulate the migration of immune cells. 3) The gut microbiota may translocate to distant tumor sites and alter their immune response or therapeutic efficacy. Immune cells in the gut-associated lymphoid tissue (GALT) can migrate to distant tumors under conditions of sensitive microbial signaling and perform immune-stimulating or inhibitory functions. 4) Bacterial metabolites, antimicrobial peptides, and bacteria can induce dendritic cells (DC) to migrate to lymph nodes to stimulate T cells and B cells. Microbe-associated molecular patterns (MAMPs) can regulate innate immunity by signaling through pattern recognition receptors such as TLRs.

The TME is the environment in which tumors grow, and it consists of blood vessels surrounding tumor cells, immune cells, fibroblasts, bone marrow-derived inflammatory cells, different signaling chemicals, and extracellular matrix. The TME can regulate tumor growth, promote tumor invasion and metastasis, mediate tumor immune escape, and promote or weaken the carcinogenic process (180–183). The TME is a complex system that includes many different types of cells, abnormal blood vessels, and immune-suppressive cytokines and is one of the important reasons for tumor evasion of immune surveillance (184) (**Figure 4**).

Mononuclear phagocytes (MPs), including monocytes (Mo), macrophages (Mac), and DCs, are the main innate immune cells and an important component of the TME. A recent study revealed the influence of the microbiota on MPs in the TME and innovatively proposed that MPs in the TME can be reshaped by microbiota to enhance the efficacy of immune checkpoint inhibitors (ICIs) (185). As sequencing technology advances, it is now known that various bacteria colonize human tumors, proliferate, and regulate immune function within tumors. The possible mechanism is that these bacteria can selectively settle in tumors that have a rich blood supply and relatively leaky vascular systems through the potential chemotactic gradient of necrotic cell debris. Once settled, they can selectively thrive in the relatively hypoxic TME (especially anaerobic or facultative anaerobic bacteria) (186).

Studies have shown that each cancer subtype has a unique microbiome with specific metabolic functions, and bacteria within tumors mainly exist in cancer cells and immune cells (187, 188). Therefore, the microbiome within tumors plays a crucial role in the development and treatment of tumors (69, 189–191).

The pancreas was once thought to be sterile, but new evidence shows that the microbiota within tumors can affect the progression and treatment of pancreatic cancer (48, 192). Researchers found that in 86 out of 113 tested human PDACs, representing 76% of the tumor tissues, there were bacteria present, mainly Gammaproteobacteria, which can metabolize the chemotherapeutic drug gemcitabine (2’, 2’-difluorodeoxycytidine) into its inactive form, 2’, 2’-difluorodeoxyuridine.

Resistance to gemcitabine is produced within tumors by Gammaproteobacteria, is dependent on bacterial cytidine deaminase (CDD_L_) expression, and can be removed by cotreatment with the antibiotic ciprofloxacin in a mouse model of CRC (48). Aykut et al. (50) found that *Malassezia* spp. were abundant in both genetically engineered mouse models and human pancreatic tumors, and that fungal or fecal bacterial transplantation selected from mice carrying PDAC accelerated tumor progression.

In addition, Riquelme et al. (193) found that the abundance of *Pseudoxanthomonas*, *Saccharopolyspora*, and *Streptomyces* spp. in tumors was highly predictive of long-term survival in pancreatic cancer patients, and a more diverse tumor microbiome composition was observed in long-term survivors, which may be attributed to the diverse microbiome promoting recruitment and activation of CD8^+^ T cells for an antitumor immune response.

According to research, the microbiota alters the immunological microenvironment of the lungs, promoting tumor formation. The resident immune cell network of the lung maintains lung tissue homeostasis while also providing immunological defense against invading infections (194). The development of lung cancer is closely associated with chronic inflammation, characterized by the infiltration of inflammatory cells and the accumulation of pro-inflammatory cytokines (such as cytokines, prostaglandins, and chemokines), which can stimulate various processes, including cell proliferation, angiogenesis, and metastasis (195, 196). Previous studies have shown that the local microbiome can activate lung-resident γδ T cells to cause lung adenocarcinoma-related inflammation. GF or antibiotic-treated mice have significant protective effects against KRAS mutation and p53 loss-induced lung cancer development. Mechanistically, commensal bacteria stimulate bone marrow cells to produce Myd88-dependent IL-1β and IL-23, inducing the proliferation and activation of Vγ6^+^Vδ1^+^γδ T cells that produce IL-17 and other effector molecules to promote inflammation and tumor cell proliferation (42).

Th2 cells and innate lymphoid cells 2 (ILC2) can stimulate tumor growth by secreting cytokines such as IL-4, IL-5, and IL-13. The oncogene Kras-G12D increases the expression of IL-33 in PDAC cells, and fungi in PDAC tissue can drive IL-33 secretion, further recruiting and activating Th2 cells and ILC2 in the tumor, ultimately inhibiting the antitumor immune response and promoting tumor progression (52).

In immune cells, neutrophils and Tregs are key cells for cancer growth and development (197–201). Neutrophils can activate the interaction between cancer cells and endothelial cells in the primary TME, thereby promoting tumor metastasis (202). Neutrophil cytokines, chemokines, growth factors, and serine proteases create a milieu that promotes tumor growth. Tumors control neutrophil differentiation in the early stages to produce multiple phenotypic and functionally polarized states that can affect tumor behavior (203). In melanoma, neutrophils recruited by TLR4 signaling can induce cancer cells to migrate to endothelial cells, promoting cancer metastasis (204). The number of neutrophils in peripheral blood and tumor tissue of individuals with various forms of cancer has increased. Importantly, these findings link neutrophils to worse clinical outcomes in cancer patients, implying that these cells may play a role in tumor promotion. In fact, many *in vitro* and *in vivo* functional studies have shown that tumor-stimulated neutrophils promote angiogenesis and immune suppression, as well as the migration, invasion, and metastasis of tumor cells (203, 205). However, the enrichment of gut microbiota reduces the number of neutrophils in circulation. One study reported that muscle atrophy in mice fed with *Lactobacillus reuteri* resulted in reduced systemic inflammation, better tumor suppression compared to the control group, and reduced numbers of neutrophils in the blood (206).

Tregs are essential for maintaining the balance of the immune system and balancing beneficial inflammatory responses during infection (207, 208). Tregs regulate host immune responses aggregated near the TME, inhibit antitumor inflammatory responses, and counteract antigen-specific effector T-cell responses (209). The TME promotes the differentiation and proliferation of Tregs and the secretion of immune-suppressive factors, thereby promoting immune suppression in tumor tissue (210). According to research by Arpaia et al., thymic-independent Treg production is stimulated by SCFAs called butyrate produced by symbiotic microbes during starch fermentation. Increased extrathymic differentiation of Tregs is the cause of the rise in Treg number following butyrate administration (82). In addition, based on animal model data, the Chinese herbal formula YYFZBJS decoction can regulate the natural gut flora, including *B. fragilis* and *Lachnospiraceae*, and prevent and inhibit the development of intestinal tumors by reducing the accumulation of CD4^+^ CD25^+^ Foxp3^+^ Tregs in the lymph nodes and MLNs of Apc Min/^+^ mice (211).

Based on the close interaction between the host microbiome and immune response in the TME, some scholars believe that regulating the gut microbiome to treat tumors is a feasible anticancer treatment strategy (164). The TME is impacted by the ongoing and positive interaction between the gut microbiome and microbial metabolites in the TME. This interaction affects IECs and host immunology, promoting or preventing the development of tumors (212). Ma et al. (213) analyzed the microbial composition of bacteria within prostate cancer tumors to determine the impact of the microbiome on prostate cancer metastatic growth. They identified microbial communities such as *Listeria monocytogenes*, *Methylobacterium radiotolerans JCM 2831*, *Xanthomonas albilineans GPE PC73*, and *Bradyrhizobium japonicum* that could effectively prevent prostate cancer growth (213).

In conclusion, the gut microbiome and tumor immunity are inextricably interconnected. The gut microbiome’s antitumor immune action requires additional investigation, even though the precise mechanism is still unclear in the modern era of tumor immunotherapy.

### 3.3 The impact of gut microbiota on tumor immunotherapy

Numerous studies have shown that antitumor therapies such as chemotherapy, radiation therapy, and immunotherapy can alter the gut microbiome of patients (214). Using the immune system for defense against cancer is a technique known as cancer immunotherapy. ICIs, oncolytic virus therapy, cancer vaccines, cytokine therapy, and adoptive cell transfer (ACT) are the main categories of current immunotherapies (215–223). Their common feature is to enhance the immune response, including innate immunity and/or adaptive immunity to clear cancer cells. Compared with other antitumor therapies, immunotherapy can significantly improve the survival rate of cancer patients. Nonetheless, there are still many patients who cannot benefit from immunotherapy because many factors, such as programmed cell death protein 1 ligand 1 (PD-L1) expression and tumor mutation burden, limit the response of many patients to immunotherapy (224).

Various gut microbiomes also play a regulatory role in cancer treatment. Microbial communities and their metabolites provide key signals for the development and function of the host immune system (174, 225–230). Using synthetic biology techniques, Canale et al. (231) created an engineered probiotic strain of *Escherichia coli Nissle 1917*. Its colonization in tumors raised the amount of L-arginine there, increased the number of tumor-infiltrating T cells, and significantly boosted tumor clearance when combined with PD-L1-blocking antibodies. The research discovered that these bacteria’s antitumor effects were mediated by L-arginine and were reliant on T cells (231).

The regulation of the gut microbiome on immune therapy response provides a new possibility for cancer treatment (**Figure 3**). Previous studies have shown that the gut microbiome may be a predictive biomarker of immunotherapy efficacy. The higher the diversity of the gut microbiome, the longer the objective response rate and survival rate of immunotherapy (232).

As early as 1813, it was reported that natural bacterial infections could be used as drugs against malignant tumors. Vautier reported that the tumor of a cancer patient with gas gangrene disappeared (224, 233). Fehleisen (1883) and later William B. Coley tested the live infection factor of dengue fever (later known as group A Streptococcus or pyogenic streptococcus) as a means of treating cancer (234–236).

Emerging evidence suggests that the gut microbiome can regulate antitumor immunity through various mechanisms (237). For example, certain antitumor microorganisms such as *Bacteroides thetaiotaomicron* and *B. fragilis* can activate DCs through TLR-4 signaling, promote Th1 and cytotoxic CD8^+^ T-cell responses, and help tumor immune surveillance and eradication (238). Bifidobacteria produce inosine, which enhances the cytotoxic activity of CD8^+^ T cells by agonizing adenosine 2A receptor signaling in T cells (239).

Although resident bacterial communities exist in the extraintestinal organs of healthy individuals, in cases of inflammation, the intestinal barrier permeability is further increased, enhancing the translocation of bacteria and bacterial components from the intestine to distant sites (240, 241). Bacterial translocation is not always associated with protumor inflammation but may also be associated with enhanced antitumor immunity. Cyclophosphamide induces immunogenic cell death in cancer cells and promotes the differentiation of Th17 and Th1 cells, thereby enhancing treatment efficacy (242, 243). Cyclophosphamide also causes increased intestinal permeability and bacterial translocation from the intestine to lymphoid organs. Surprisingly, bacterial translocation of Gram-positive bacterial species induced by cyclophosphamide results in the production of Th1 memory T cells and the differentiation of Th17 cells producing IFN-γ, which is crucial for antitumor immune responses during treatment (122, 244).

Radiation therapy can treat most tumors whether used alone or in combination. Increasing evidence shows an interaction between radiation exposure and the human intestinal microbiota. After radiation therapy, the structure and composition of the microbiota are directly altered, such as by the decrease in the relative abundance of beneficial microbiota such as *Bifidobacterium* and *Faecalibacterium* (245–247). When radiation therapy is used to treat tumors in the abdomen and pelvis, radiation may damage intestinal mucosal barrier function, affecting food absorption, and even causing immune changes (248–251). However, there is an interaction between radiation therapy and microbiota. Although Radiation therapy (RT) treatment leads to an imbalance in the intestinal microbiota, these changes in the microbiota may be an important determining factor for the effectiveness of radiation therapy against tumors (252).

Microbes have the potential to serve as biomarkers of response to ICIs (253). In melanoma, non-small cell lung cancer, urothelial carcinoma, and renal cell carcinoma, the impact of the gut microbiota on the efficacy and interactions of ICIs has been documented. By examining the oral and gut microbiome of 112 melanoma patients undergoing anti-Programmed Cell Death Protein 1 (PD-1) immunotherapy, significant differences in the diversity and composition of gut microbiomes were observed between responders and nonresponders, and when analyzing the patients’ fecal microbiomes, microbial α-diversity (P < 0.01) and the relative abundance of tumor Clostridiales (P < 0.01) were significantly higher. Transplanting fecal microbiota from patients with a favorable response to ICIs into GF mice enhanced their antitumor immunity (164). Mager et al. (239) found that *Bifidobacterium pseudolongum*, *Lactobacillus johnsonii*, and *Olsenella* can increase the efficacy of ICIs 4-fold in four cancer mouse models.

Resistance to ICIs targeting the PD-1/PD-L1 axis induces sustained clinical responses in a considerable proportion of cancer patients (254). We found that the main resistance to ICIs could be attributed to abnormal gut microbiota composition. Antibiotics inhibit the clinical benefits of ICIs in advanced cancer patients, and transplantation of fecal microbiota (FMT) from cancer patients who respond to ICIs into GF or antibiotic-treated mice can improve the antitumor effect of PD-1 blockade, whereas FMT from nonresponders cannot improve the efficacy of PD-1 (255).

*Akkermansia muciniphila* is a gut bacterium that has been shown to be associated with systemic effects on host metabolism and PD-1 checkpoint immune therapy and induces immunoglobulin G1 (IgG1) antibodies and antigen-specific T-cell responses in mice (256). The relative abundance of *A. muciniphila* was found to be correlated with the clinical response to ICIs in patients’ fecal samples as revealed by metagenomics at diagnosis. Oral supplementation of *A. muciniphila* restored the efficacy of PD-1 blockade in mice in a white blood cell-dependent manner by increasing the recruitment of CCR9^+^ CXCR3^+^ CD4^+^ T lymphocytes to the mouse tumor bed after FMT using nonresponder feces (229). Preliminary studies in mouse models have identified the role of the gut microbiota in supporting the efficacy of CpG oligonucleotide immunotherapy and immunostimulatory cyclophosphamide chemotherapy, and further research has demonstrated the immunostimulatory effects of specific bacteria such as *Bifidobacterium* and fragile Bifidobacteria that enhance the efficacy of ICIs in mouse models (115, 257). An experiment conducted at the University of Pittsburgh evaluated early data on the use of FMT combined with pembrolizumab to treat melanoma patients who had failed anti-PD-1 therapy. The report indicated that two out of three patients experienced stable disease or tumor regression (258). Another study evaluated FMT in patients with melanoma who had become resistant to PD-1 inhibitor therapy. One patient experienced illness regression after the initial scan, while another had a considerable reduction in disease load and lived for an additional 8 months. These patients’ tumor histology revealed increased immune cell infiltration, and sequencing of their gut microbiome revealed alterations in the bacterial population (259).

Peng et al. performed 16SrRNA testing on fecal samples from 74 patients with advanced gastrointestinal tumors who received anti-PD-1/PD-L1 therapy. Before and during treatment, the patients who had higher proportions of *Prevotella fungi bacteria* in their fecal microbiota showed better PD-1/PD-L1 treatment responses and longer progression-free survival. Patients with higher relative abundances of *Prevotella*, *Rumatococcaceae*, and *Trichophyton* spp. had better treatment responses, and gut bacteria that produce SCFAs, including lactobacilli and streptococci, were positively correlated with the response to anti-PD-1/PD-L1 therapy in different types of gastrointestinal tumors. Microorganisms are potential response markers for ICIs (253). Bullman et al. found that human primary colon adenocarcinoma xenografts in mice retained living clostridial bacteria and their associated microbiota through consecutive passages. Treatment of mice carrying colon cancer xenografts with the antibiotic metronidazole reduced the clostridial load, cancer cell proliferation, and overall tumor growth. These findings support further investigation into antibacterial interventions as potential treatment methods for clostridial-related colon cancer patients (260). Tumor response rates and survival rates of Immune Checkpoint Blockade (ICB) cancer patients decreased when they received antibiotics during treatment (261). Routy et al. (229) found that if patients received antibiotics early, either before or after ICB treatment, their survival rates were significantly reduced. In a mouse model of melanoma, it was found that oral administration of Bifidobacteria alone can improve tumor control to the same extent as treatment with PD-L1-specific antibodies, and combined therapy almost eliminates tumor growth (230). The activation of antigen-presenting cells and increased cytotoxic T lymphocyte infiltration into the tumor may be the mechanisms underlying the improved antitumor response, although it is still unknown if microbiota-regulated CD4^+^ T cells may also stop tumor progression. According to studies, distinct species of *B. thetaiotaomicron* are required for Cytotoxic T-Lymphocyte-Associated Protein 4 (CTLA-4) inhibitors to exert antitumor effects. In mice and patients, specific T-cell responses to *B. thetaiotaomicron* or *B. fragilis* are correlated with the efficacy of CTLA-4 inhibitors, and antibiotic-treated or GF mice do not respond to CTLA inhibitors, which can be overcome by administering *B. fragilis*, immunizing with fragilis polysaccharides, or transferring fragilis-specific T cells (238). Recent studies have shown that the use of vancomycin can enhance the efficacy of Chimeric Antigen Receptor T-cell therapy (CAR-T) therapy in mouse models of cervical cancer. Mechanistically, vancomycin treatment induces an increase in systemic CD8α^+^ DCs, elevating IL-12 levels and maintaining the efficacy of systemically transferred antitumor T cells (262). The gut microbiota’s metabolites, like SCFAs, may also influence tumor immunotherapy, according to mounting data. One study showed that butyrate and propionate enhance the antitumor activity of Cytotoxic T Lymphocyte (CTL) and CAR-T cells through metabolic and epigenetic reprogramming, increasing the production of effector molecules such as CD25, IFN-γ, and TNF-α (263). Researchers revealed that they could dramatically improve the therapeutic impact of PD-1 mAb through providing mice oral doses of pectin, inulin, and other polysaccharide dietary fibers. Adding these prebiotics can increase the relative abundance of key symbiotic microbes, such as *Akkermansia* and *Lactobacillus*, and SCFAs, further promoting CD8^+^ T-cell infiltration into the tumor (264). In addition, SCFAs have been found to enhance the memory potential of antigen-induced CD8^+^ T cells and trigger their differentiation into stem-like Tcf1^+^PD-1^+^CD8^+^ T cells, which produce effective and long-lasting antitumor effects (265).

The exploration of how gut microbiota regulates the efficacy of immune therapy is proposed by Mager et al. (239) Gut microbiota may regulate the outcome of immune therapy by stimulating or inhibiting possible mechanisms of tumor immunity, such as bacterial metabolites entering the bloodstream and regulating the gene expression of various cells; regulation of innate immunity by pattern recognition receptors; complete live bacteria possibly transferring to distant tumors and affecting the immune response or drug activity; immune cells regulated by microbial signals in GALT can migrate and play immunostimulatory or immunosuppressive functions in distant tumors; and cytokines may be released through corresponding microbial stimulation in GALT, which could possibly enter the circulatory system and regulate the downstream immune function (173).

The effectiveness of immune therapy is significantly impacted by immunological resistance, and immune therapy susceptibility is correlated with gut microbiota. In addition to contributing to immune therapy for tumors, the gut microbiota may also affect immune resistance. *Clostridium* may play a role in patient resistance to chemotherapy, and a large-scale study showed that the abundance of *Clostridium* is associated with a decrease in overall survival (OS) (266). TLR4, which is expressed on CRC cells, is activated by Clostridia, making these tumor cells more resistant to oxaliplatin-induced cell death. This results in treatment failure and encourages chemotherapy resistance in CRC patients (72). Some strains of lactic acid bacteria, such as *Lactobacillus fermentum*, are also believed to weaken the response to immunotherapy (257). In addition to mediating therapeutic effects, the gut microbiota can also regulate the toxic effects of tumor treatment. The differences in gut microbiota composition are associated with graft-versus-host disease (GVHD) in different hematologic malignancies undergoing allogeneic stem cell transplantation (267–269).

Radiation therapy is an effective method for treating tumors. The interaction between gut microbiota and radiation therapy is bidirectional (246). Radiation therapy can disrupt the composition of the gut microbiota, which can have positive or negative effects on the efficacy of tumor treatment. Typically, this disruption manifests as a decrease in the abundance and diversity of the gut microbiota, an increase in harmful microbial populations (such as Proteobacteria and Clostridia), and a decrease in beneficial microbial populations (such as Firmicutes and Bacteroidetes) (245, 270).

Preclinical evidence suggests that patients exposed to broad-spectrum antibiotics may experience a reduction in the effectiveness of cancer radiotherapy. Changes in the gut microbiota caused by antibiotics may be a key factor contributing to this phenomenon (271). Another study found that whole-body irradiation enhanced the translocation of gut bacteria to the MLNs in a melanoma mouse model, resulting in a stronger anticancer response (272). Although increasing evidence suggests that the human gut microbiota has radioprotective effects, further research and exploration are needed to understand how the gut microbiota influences the response to radiation therapy.

## 4 Summary and outlook

The broad concept of microbiota extends beyond the gut microbiota and encompasses the microecology of various tissues within the body and even external ecological environments, forming a three-dimensional spatial microbiota system that profoundly influences the occurrence of diseases and the body’s resistance. There is an inseparable relationship between gut microbiota and tumors and tumor immunity. Imbalanced microbial ecology can induce tumor formation, and tumor formation can also cause microbial ecology disorders. The gut microbiota has undergone distinct modifications in various tumors, and these changes can be used as biomarkers for supplementary tumor diagnosis. The benefits brought about by utilizing probiotics in tumor immunotherapy are questionable, since the process by which microbial communities in the TME affect tumor progression is complex and mysterious. Some probiotics may hinder the effect of immunotherapy and even promote cancer progression. Although the specific mechanism is yet to be elucidated, the antitumor immune function of gut microbiota is worth further exploration in the current era of tumor immunotherapy. FMT has shown potential in tumor immunotherapy because of its effect on the microbiome (135, 273–276). Currently, FMT is being utilized more frequently beyond the treatment of metabolic syndrome, diabetes, Crohn’s disease, Parkinson’s disease, multiple sclerosis, psoriasis, anorexia nervosa, or Alzheimer’s disease (277–280). However, FMT still has many side effects, such as abdominal discomfort, cramps, bloating, diarrhea, or constipation, and its effect varies among individuals due to different microbial community compositions, as well as various factors such as age, diet, and medication (281). In addition, bacteriophages’ specific killing of gut microbiota provides direction for the specific elimination of microorganisms that promote tumor development and hinder microbial translocation for various reasons, as well as improving the efficacy of tumor treatment (282). Of course, the uncertainty introduced by these methods raises concerns about the role of the microbiota in tumor immunotherapy.

The interaction between microbial species, number, metabolites, and immune cells is one area that will require further investigation in order to fully understand the relationship between gut microbiota and immunotherapy. To create more individualized treatment plans, it is also essential to look into how different types of tumors are impacted by gut bacteria. In future studies, it is also important to consider the influence of an individual’s genetic background and lifestyle on gut microbiota to better understand the interaction between microbiota and tumor therapy. The findings in this paper will contribute to the development of novel tumor treatment plans, increased immunotherapy effectiveness, and decreased side effects.

In the future, the diagnosis and treatment of diseases will gradually transition from anti-disease, antibacterial, and antitumor drugs to comprehensive monitoring of the immune function. The evaluation of the comprehensive immune function is based on the balance of the overall and local dynamic microbiota, and the gut and skin are all components of the microbiota and places for maintaining normal immune function. Therefore, from the perspective of maintaining a normal comprehensive immune function, maintaining the balance of local microbiota is a prerequisite for maintaining overall balance, and the cross-dialog of different local microbiota forms a good symbiotic system. This kind of symbiosis is the core area for exploring the nature of health and the origin of diseases. Immunology’s advancements have embraced traditional Chinese and Western medicine in separate directions but ultimately to the same cause, creating a brand-new area of medicine. The evaluation of the comprehensive immune function must be carried out from the most fundamental perspective and presented in a digital immune force method, from mild regulation to dynamic detection of immunological normalization. This is also a trend in the evaluation of diagnosis and treatment and drug development and is one of the important trends in the development of precision immunology. It is also an important trend in the development of aging biology.

## Author contributions

All authors listed have made a substantial, direct, and intellectual contribution to the work, and approved it for publication.
